# Do “central sensitization” questionnaires reflect measures of nociceptive sensitization or psychological constructs? Protocol for a systematic review

**DOI:** 10.1097/PR9.0000000000000962

**Published:** 2021-10-22

**Authors:** Greig R. Adams, Wiebke Gandhi, Richard Harrison, Carien M. van Reekum, Ian Gilron, Tim V. Salomons

**Affiliations:** a School of Psychology and Clinical Language Sciences, University of Reading, Reading, United Kingdom; b Department of Psychology, Queen's University, Kingston, ON, Canada

**Keywords:** Central sensitization, Central Sensitization Inventory, Pain Sensitivity Questionnaire, Quantitative sensory testing, Emotional sensitization, Nociceptive sensitization

## Abstract

**Introduction:**

Central sensitization (CS) was first defined in animal studies to be increased nociceptive responsiveness due to sensitization of neurons in the central nervous system, usually the result of prolonged nociceptive input or a disease state. Recently, the concept of CS has been adopted in clinical assessments of chronic pain, but its diagnosis in humans has expanded to include the enhancement of a wide range of nociceptive, sensory, and emotional responses. Many poorly understood pain disorders are referred to as “central sensitivity syndrome,” a term associated with a broad range of hypervigilant sensory and emotional responses. Diagnosis often involves a review of medical records and an assessment of behaviour, emotional disposition, and overall sensitivity of a patient. Obviously, these assessments are unable to directly capture the responsiveness of nociceptive neurons. The purpose of this review is to ascertain whether self-report questionnaires associated with central sensitization and the diagnosis of central sensitivity syndrome are associated with enhanced nociceptive responses or whether they more validly measure sensitivity in a broader sense (ie, including emotional responses).

**Methods:**

Following the PRISMA guidelines, a detailed search of studies that involve the Central Sensitization Inventory or Pain Sensitivity Questionnaire correlated with either nociceptive sensory tests (quantitative sensory testing) or emotional hypervigilance (anxiety, depression, stress, etc) will be conducted on MEDLINE, PsychINFO, and Web of Science.

**Perspective:**

The review is expected to synthesize correlations between sensitivity questionnaires and nociceptive or emotional sensitivity to determine whether these questionnaires reflect a broadened understanding of the term “central sensitization.”

## Abstract 2

A protocol for a systematic review examining how central sensitisation questionnaires correlate with sensory or psychological measures to clarify the use of “central sensitisation” in clinical contexts.

## 1. Introduction

Central sensitization (CS) is defined by the International Association for the Study of Pain as “increased responsiveness of nociceptive neurons in the central nervous system to either normal or subthreshold afferent input.”^8^ This definition was derived from animal studies that explore hypersensitivity to stimuli,^10^ greater responsiveness to non-noxious stimuli,^12^ and increased pain response evoked by stimuli outside the area of injury.^4,10,12^ It has been linked with a multitude of chronic pain disorders in humans.^9,11,15,24,26,34^ The association between CS and chronic pain seems to include multiple mechanisms involving spinal cord hyperexcitability^21^ and ascending or descending modulatory systems.^29^ As of yet, there is no conclusive method of establishing the presence of CS in humans, although quantitative sensory testing (QST) is used to assess the dynamic modulation of nociceptive signals, which can suggest the presence of CS.^3^

Although QST allows for a comprehensive assessment of pain sensitivity profiles, it often involves select training, expensive laboratory equipment, and sufficient time where the patient's presence is required in the laboratory which makes testing difficult at a clinical level.^23^ Self-report questionnaires would make a pragmatic alternative assessment of CS in clinics, allowing for quick and convenient assessment at little cost. To serve this purpose, however, these questionnaires would need to demonstrate acceptable associations with known measures of CS to show sufficient construct validity.

Two such self-report questionnaires that are widely used in the assessment of CS and pain sensitivity are the Central Sensitisation Inventory (CSI) and the Pain Sensitivity Questionnaire (PSQ). The CSI was designed to identify patients who have symptoms that may be related to CS or central sensitivity syndromes (CSSs), such as fibromyalgia, neck injury, temporomandibular joint disorder, or migraine or tension headaches.^19^ It has been shown to be a reliable and valid psychometric instrument for identifying individuals vulnerable to pain.^13^

One question that arises regarding the inventory, however, is the degree to which it reflects CS as defined by the animal literature, ie, increased summation of nociception, reduced inhibition of pain, hypervigilance to noxious and non-noxious stimuli (allodynia or hyperalgesia), and widespread pain sensitivity.^10,31,32^ Instead, the questionnaire seems to focus more on hypersensitivity in a broader sense including feelings of anxiety and depression, as well as cognitive impairment. If this is the case, it may contribute to a broadening usage of the term “central sensitization” to include particular psychological profiles rather than simply centrally enhanced nociceptive responsivity.

Central sensitivity syndrome (CSS) is a recently developed diagnosis for several pain conditions,^33^ which may embody this construct drift. Central sensitivity syndrome describes a group of medically unexplained disorders (eg, fibromyalgia or chronic fatigue) for which CS may be a common underlying cause. Because of the difficulty of directly assessing the presence of CS in humans, the diagnosis of CSS includes a focused review of medical records, interviewing techniques, and observations of pain and emotional behaviour, mental fog, drug and food intolerance, psychiatric disorders, and trauma.^5^ The CSI was developed as a self-report screening instrument to help identify patients with CSS.^13,19^ Although this measure has demonstrated clinical utility^13,19^ in identifying individuals vulnerable to pain, a question remains as to whether this diagnostic tool truly reflects the traditional understanding of “central sensitization” as portrayed by early animal studies^10^: increased responsiveness of nociceptive neurons in the central nervous system. We are interested in exploring the extent to which “central sensitivity syndromes,” diagnosed using the CSI, may not truly represent the CS mechanism derived from preclinical animal studies. A diagnosis of CSS gives a central mechanism to legitimize a patient's pain and therefore may have clinical value, yet this diagnosis may not truly be representative of the canonical (preclinical) mechanism of CS as the questionnaires title would suggest. Instead, the questionnaire may reflect a broader definition of sensitivity which includes depression, anxiety, stress, and neuroticism.

The PSQ may more directly measure the sensory facilitation involved in CS.^25^ It focuses more on respondents imagining situations that involve nociceptive input and predicting how they would react. The questions are posed to measure sensitization to sensory input, but the degree to which it reflects a top-down component influenced by personality type or disposition remains open. More specifically, whether it reflects these psychological profiles to the same degree as the CSI is a germane question for understanding how closely related and psychometrically distinct these measures are.

In this review, we aim to evaluate the degree to which measures of nociceptive sensitization (eg, quantitative sensory measures) correspond to these sensitivity questionnaires (CSI and PSQ) and how strongly they are influenced by psychological factors. If the latter is a strong determinant of scores on these measures, inferring “central sensitization” on the basis of such scores would represent a broadening of the definition of CS (construct drift) to include hypersensitivity to psychological states such as depression and anxiety. If this were the case, it might necessitate reconsideration of what is meant by the term “central sensitization” in clinical contexts and perhaps questioning of the presumption of a specific physiological mechanism on the basis of such measures.

## 2. Objective

The objective of this overview is to review the available studies that examined the association between questionnaires putatively measuring CS (the PSQ or CSI) against quantitative sensory measures and psychological questionnaires. In doing so, we aim to assess and compare the 2 questionnaires in the degree to which they assess nociceptive sensitization or emotional sensitization. Our main aim is to assess whether the CSI is likely to reflect responsiveness of nociceptive neurons or whether it assesses sensitization in a broader context. We are also interested to assess whether the PSQ more accurately maps on to the preclinical definition of CS.

### 2.1. Hypotheses

Given the nature of the questions posed in the CSI and PSQ, we expect that the CSI will be strongly correlated with psychological constructs such as depression, anxiety, stress, and neurotic behaviour. As the PSQ focuses on questions related to pain sensitivity, we expect the PSQ to be more strongly map on to measures of nociceptive stimulation (thermal, pressure, and electrical) such as pain thresholds, pain tolerance, and temporal summation. We are also interested in exploring the extent to which self-report questionnaires may reflect descending aspects of modulation (impaired inhibition), therefore including CPM as a measure of interest*.*

## 3. Methods

This protocol is developed in accordance with the PRISMA-P guidelines^16^ and is registered in the PROSPERO register (CRD42021208731).

### 3.1. Sources of evidence

We will conduct a detailed search on MEDLINE, PsychINFO, and Web of Science from their inception until the date of the search. Two separate searches will be conducted (see search terms in section below), and some results are expected to overlap (duplicates will be removed). Both searches will include the CSI or the PSQ. One search will review the CSI or PSQ for sensory correlates (eg, QST measures), and the second search will review their correlations with psychological questionnaires (eg, anxiety, depression, pain catastrophising, etc) as well as any personality questionnaires that suggest neurotic behaviour. We are specifically interested in neuroticism given the evidence that it is related to pain sensitivity^20^ but have cast a broader scope to examine whether other personality factors are relevant. We will also review the bibliographies of any relevant study identified and search Google Scholar to identify any additional published articles.

### 3.2. Search terms

#### 3.2.1. Search 1 terms

“Quantitative sensory testing” or “wind-up” or “temporal summation” or “conditioned pain modulation” or “pain threshold” or “pain ratings” or “hyperalgesia” or “allodynia” or “offset analgesia” or “widespread pain” or “pain tolerance” or “evoked pain” or “experimental pain” AND “central sensitization inventory” or “CSI” or “PSQ.”

#### 3.2.2. Search 2 terms

“Depression” or “anxiety” or “stress” or “catastrophizing” or “rumination” or “neuroticism” or “personality” or “abuse” or “trauma” AND “central sensitization inventory” or “CSI” or “PSQ.”

### 3.3. Inclusion criteria

Studies eligible for inclusion in the review will include human studies only. They must be written in English and must be an original peer-reviewed experiment (ie, not a dissertation, case study, or review article). Finally, studies must include at least one of the CSI or PSQ instruments. The CSI or PSQ must be correlated against at least 1 psychological or sensory measure of interest.

### 3.4. Report selection

#### 3.4.1. Types of studies

The review will include studies that correlate the PSQ or CSI with sensory or psychological measures. In studies that involve an intervention, measures will only be considered if they were assessed before the intervention.

#### 3.4.2. Types of participants

We will include studies with participants of all ages. Healthy subjects as well as studies involving patients with chronic pain will be included. Where comparative studies are available, we will compare the findings for patients with pain against healthy controls.

### 3.5. Data collection, extraction, and management

Two independent reviewers will assess studies for eligibility. Initially, titles and abstracts will be screened, and full-text screening will be performed on citations believed to be potentially eligible. Any discrepancies between the reviewers will be resolved by discussion and consensus. A third reviewer will be consulted in cases of disagreement.

One reviewer will extract relevant data from each study (r co-efficient and *P* value). If these values are not given within the article, authors will be contacted to provide the relevant correlation co-efficient and *P* values. The second reviewer will check the extracted data.

### 3.6. Outcome measures

Our primary outcomes will include the CSI or the PSQ correlated with sensory measures associated with CS. These include QST measures, temporal summation, conditioned pain modulation, pain thresholds and tolerance, and any measure related to nociceptive hypersensitivity or widespread pain.

The second primary outcome measure will include the CSI or the PSQ correlated with psychological factors associated with CS. These include questionnaires that assess depression, anxiety, stress, pain catastrophizing, abuse, trauma, mindfulness, neuroticism or personality, and any other measure related to emotional hypersensitivity. In studies that report trait and state levels for a construct, ie, the state-trait anxiety questionnaire, we will try to differentiate between the 2 levels if our search finds enough data to make analysis of this distinction reliable. However, given that these measures have high intercorrelation,^22^ we will collapse scores into an average correlation score for that construct if there are not sufficient data.

### 3.7. Analysis plan

#### 3.7.1. Analysis of outcomes

Meta-analyses will be conducted for CSI correlations with psychological constructs such as depression, anxiety, or catastrophising when measures of the same construct (eg, different anxiety questionnaires) are judged to be similar enough to support valid conclusions.

Meta-analyses for quantitative sensory measures will also be conducted by analysing each measure separately: Pressure pain threshold, heat pain threshold, conditioned pain modulation, temporal summation, etc will be separately meta-analysed against CSI. Any other findings with insufficient data for a meta-analysis will be presented as a narrative review with a descriptive analysis.

The same meta-analyses will be conducted for the PSQ.

Assessing the 2 questionnaires (the CSI and PSQ) for what correlations they map on to will allow us to assess what these questionnaires are measuring. Comparing the 2 questionnaires against each other will allow us to make relative comparisons accounting for mono-method bias (ie, will both questionnaires be strongly correlated with psychological measures as they are both largely based on self-reports?). We will test to see whether the correlations are significantly different and therefore will be able to assess whether one questionnaire more strongly correlates with emotional sensitization and whether the other is a more valid measure of nociceptive sensitization.

#### 3.7.2. Analysis of risk of bias

A modified version of the quality appraisal process proposed by Hayden et al.^7^ will be conducted by 2 independent authors to evaluate potential sources of bias across 5 domains: participation bias, publication bias, attrition, methodological quality, and statistical analysis. We will assess the following for each study: (1) Were potential sources of participation bias considered and addressed? (2) Was there any missing data regarding the variables of interest? (3) Was the methodology of the variable of interest of a standardized quality? (4) Was the desirable statistical analyses performed? (5) Was the sample size adequate? Each category will be assigned a low, unclear, or high risk of bias and presented with a “risk of bias” summary. Disagreements between reviewers will be resolved with discussion and consensus. If necessary, a third reviewer will be consulted.

#### 3.7.3. Analysis of participation bias

Specific sources of participation bias will be identified, ie, age and sex characteristics of the population sample, as well as population samples that excluded participants based on mental health, physical health, or medication being taken.

## 4. Discussion

We will assess whether self-report questionnaires (the CSI and PSQ) which are widely considered to be measures of CS primarily reflect enhanced responsiveness to nociception or a broader construct including maladaptive emotional responsivity. We will do so by comparing correlations between these questionnaires and nociceptive responsivity on one hand and emotional sensitization on the other. Both questionnaires have demonstrated clinical utility in identifying pain vulnerability,^19,30^ but if they largely reflect psychological states such as anxiety, depression, and catastrophizing, their use as measures of CS may reflect a broadening of our definition of that construct.

We know that sensitization to nociception is associated with increased vulnerability to other disorders of the central nervous system such as depression, anxiety, and stress.^1,6,14,17,27,28^ Psychological distress and sensitization to nociception have a bidirectional influence on each other.^2^ Assessments of CS, particularly the CSI, seem to have used this strong association to help determine characteristics of CS syndromes.^18,19^ Although these assessments serve great utility in a diagnostic sense, we would like to explore the extent to which these questionnaires truly assess “increased responsiveness of nociceptive neurons in the central nervous system to either normal or subthreshold afferent input”^8^ and clarify that these 2 constructs of hypervigilance (nociceptive and psychological), while strongly related, are dissociable and therefore not best treated as a single construct. Doing so runs the risk of presuming a specific (but untested) physiological mechanism whenever a particular psychological profile is present. As such, it motivates us to understand exactly what these measures are examining.

## Disclosures

The authors have no conflict of interest to declare.
